# Suggestions in the Treatment of Diseases of the Respiratory Tract

**Published:** 1907-01

**Authors:** J. F. T. Jenkins

**Affiliations:** Los Angeles, California


### CLINICAL REPORTS.

### Suggestions in the Treatment of Diseases of the Respiratory Tract.

By J. F. T. JENKINS, Ph. G., M.S., M.D., Los Angeles, California.

A MARKED advance in therapeutics has taken place in the last few years and many new remedies have been introduced which, after careful clinical tests, have been found to be vastly superior to former methods of treatment. A drug which has attracted considerable attention is the new morphine derivative, heroin. It has been brought before the profession for the purpose of allaying cough and to take the place of codeine as a more efficient substitute. Its action in relieving cough and dyspnea is much more prompt and decided, and the frequent deleterious after-effects of codeine and morphine,—nausea, vomiting, headache, constipation, gastric pains, tinnitus, and visual hallucinations,—have never been observed during its administration. Results are equally as good with children as with adults, and it has now taken a permanent place in the armamentarium of the physician.

Some time after the introduction of heroin, while I was Acting Assistant Surgeon in temporary command of the station for the United States Marine Hospital Service, I had a number of government patients, sailors of the merchant marine, at the Los Angeles Infirmary (Sister's Hospital) under my professional care. In one case of persistent cough, which was extremely distressing to the patient and harassing to his friends, I tried everything I had heretofore used, without obtaining even partial relief. The then resident physician, Dr. M. M. Kannon, urged me to try a new combination of heroin with other drugs, known as Glyco-Heroin (Smith), made by Martin H. Smith Company, of New York. Acting upon this suggestion, I gave a prescription for a few ounces of this preparation to be given in teaspoonful doses every four to six hours until relieved. The good effect was immediate and pronounced, and from that time to the present I have had positive results in relieving cough that I had failed to obtain in my previous experience of a quarter of a century, in the active practice of the medical profession. Before giving it in the case mentioned I was inclined to be sceptical, notwithstanding the frequency of favorable reports in regard to it by respectable medical journals and leading professional men.

I had tried without satisfactory results the usual mixtures heroin compound, of which there are so many without merit, failing entirely to accomplish all that heroin can be shown to do in using the preparation indicated. To satisfy myself still further and to remove a doubt in my mind that this might be an exceptional case or a mere coincidence, I was induced to give it a trial in a series of selected cases of a similar character. The result was so satisfactory that I feel constrained to add my testimony to that of others and place the record of cases before the profession; first giving a brief prelude dealing with the component parts of the prescription and its applicability in the treatment of all pulmonary and laryngeal diseases; followed by the expressed opinions of some eminent medical men as to the curative action of heroin, the most important drug in this excellent combination.

Glyco-Heroin (Smith) is a true solution of heroin in glycerine; each teaspoonful represents one-sixteenth of a grain of heroin, with ammonium hypophosphite, hyoscyamus, white pine bark, balsam of tolu, with glycerine and aromatics. A glance at this formula shows a happy selection of drugs adding to the palliative effect of the heroin and each possessing decided curative properties of its own. It gives the physician an elegant pharmaceutical preparation, strictly ethical in character, a trial of which will satisfy him that it excels any single drug or combination of drugs in the materia medica. Limitation of space prevents an exhaustive consideration of the individual therapeutic virtues of the ingredients mentioned. With the exception of heroin, all are so well known that a very minute detail of their virtues is not necessary. It is important, however, to notice that the value of each seems to be increased tenfold, and the special sedative action intensified in the uniformly exact proportions adhered to by the Martin H. Smith Company in its manufacture. Referring again to the first component and most essential drug in this prescription—heroin—a recent writer says: "Though of recent date, this drug has already evinced its pronounced efficacy as a bronchial and pulmonary sedative and as an agent facilitating and promoting expectoration."

In the Boston Medical and Surgical Journal (Feb. 22, 1900), Dr. Geis pronounces heroin "an eminently satisfactory agent in loosening and expelling stagnant products from the lungs."

Dr. H. D. Fulton, in the New York Medical Journal, refers to it as a "veritable boon to the consumptive as a palliative in cough."

In the Journal of the American Medical Ass'n., Dr. W. Freudenthal states that the "irritable cough in tuberculous patients frequently yields to the administration of heroin, and this occurs in cases in which both morphine and codeine have proved inefficient." The same writer considers it useful also in asthma and pseudocroup.

Dr. W. H. Ambrose, of Cincinnati, reports as follows: "An agent like it, which diminishes the frequency of respiration and increases its force, finds a large field of application in conditions of dyspnea, as in nervous asthma and difficulty of breathing in cases of emphysema, chronic bronchitis, and especially phthisis."

Dr. E. R. Mitchell believes "it may be advantageously given in the cough and dyspnea of tuberculosis over a long period of time" while Dr. Morris Manges found it "a valuable aid in the treatment of pneumonia, alleviating the pleuritic stitches and the distressing cough.....It acted well in some cases not relieved by codeine."

A number of European authorities might also be quoted, notably Prof. Leo, who speaks of it in a German medical publication as a "specific in affections of the bronchi attended with dyspnea and in emphysema."

So much for heroin, with a great deal left unsaid which might be justly stated in its favor. The ammonia hypophosphite and hyoscyamus each speaks for itself. The peculiar virtues of white pine bark in checking night sweats and in allaying all inflammatory conditions of the bronchial mucous membrane, need only to be mentioned to be appreciated. That pleasant and palatable aromatic stimulant, balsam of tolu, together with glycerine, completes the prescription known as Glyco-Heroin (Smith), now so well established by the evidence of experience.

The clinical reports of many practitioners in widely separated parts of the world attest that this combination of remedies will quiet cough, remove dyspnea, relieve pain, aid and regulate expectoration, control night sweats, and cause the wornout pulmonary invalid to add to his weight and build up his general health.

The clinical evidence as shown by these carefully-kept records has thus demonstrated that this preparation possesses qualities of unusual merit, that it contains elements within it calculated to reach the morbid state in which the symptoms originate and to correct the predisposition to further trouble.

I have used Glyco-Heroin (Smith) in the treatment of all disorders of the breathing apparatus, including coughs, phthisis, laryngitis, asthma, bronchitis, pneumonia, and whooping cough.

In addition to my personal experience, I have reports from Dr. L. T. Holland, late coroner of Los Angeles County, and from Dr. Jos. Jauch, of this city, formerly of the Univ. of Wurzburg, Germany.

Dr. Holland says he prescribes Glyco-Heroin (Smith) in preference to every other preparation of a similar nature, particularly for the relief of cough in its various forms. He speaks of its innocuous but powerful analgesic and antispasmodic properties. He closes a brief account of the good results he has obtained by stating that it has more than fulfilled his expectations.

Dr. Jauch states that he has at last found a valuable mixture that combines the two important elements of palatability and effectiveness. He has found it agrees perfectly with the most sensitive stomach. He continues: "I have found it the most efficient agent I have yet used in subduing the hyperesthesia of the respiratory mucous membrane. I have used it successfully in the treatment of most affections of the air passages, particularly for the relief of cough, obstinate cases of bronchitis, in severe laryngeal cough, and in many distressing cases of tubercular cough of an aggravated character which has resisted all other medication."

The striking and surprisingly good results so uniformly obtained in the administration of this remedy can be fully verified by any unprejudiced trial in which it is tested. Such a trial may be made without hesitation, for notwithstanding its therapeutic advantages it possesses the virtue of absolute harmlessness. When physicians of the professional standing of Francis W. Campbell, M.A., M.D., D.C.L., L.R.C.P., London, Dean and Professor of Medicine, Univ. of Bishop's College, Montreal; and Dr. J. Leffingwell Hatch, late Professor of Laryngology in the New York Clinical School of Medicine, Pathologist to the Philadelphia Hospital and formerly Sanitary Inspector in the Marine Hospital Service, give this preparation their unqualified endorsement, their opinions founded on the actual treatment of a large number of cases, it is apparent that these positive, unlimited, and clear results must gain for this remedy a still fuller recognition, and lead ultimately to its universal acknowledgment as the best remedy of its class for the purposes indicated in these reports, and in clinical reports of prominent medical men in England and her colonies, in addition to the favorable testimony of many American physicians from Canada to Mexico and from Maine to California.

---

An Urgent Plea for the Use of Larger Doses of Antitetanic Serum for Lockjaw, with a Case in Point.—Rosa Engelmann believes that the ineffectiveness of antitetanic serum in cases of tetanus is due to insufficient doses. In the case, the history of which she reports, the writer administered the serum by both intraspinal and subcutaneous injections to the amount of from 60 c.c. to 80 c.c. daily to begin with, a total amount being 650 c.c., of which 520 c.c. was given subcutaneously, 85 c.c. subdurally, and 45 c.c. directly into the wound. After five days of this treatment, the man was comfortable, with flaccid muscles and less rigid jaw. Recovery took place in four weeks, and at the end of six weeks the patient was back at work.—*Medical Record*, September 22, 1906.

